# Changes in Acute Myocardial Infarction, Stroke, and Heart Failure Hospitalizations During COVID-19 Pandemic in Tuscany—An Interrupted Time Series Study

**DOI:** 10.3389/ijph.2022.1604319

**Published:** 2022-06-08

**Authors:** Sophie Y. Wang, Chiara Seghieri, Milena Vainieri, Oliver Groene

**Affiliations:** ^1^ Hamburg Center for Health Economics, University of Hamburg, Hamburg, Germany; ^2^ OptiMedis AG, Hamburg, Germany; ^3^ Institute of Management and Department EMbeDS, Sant’Anna School of Advanced Studies, Pisa, Italy

**Keywords:** COVID-19, SARS-CoV-2, stroke, heart failure, acute myocardial infarction, interrupted time series

## Abstract

**Objectives:** We evaluate the impact of the COVID-19 pandemic on unplanned hospitalization rates for patients without COVID-19, including their length of stay, and in-hospital mortality, overall, and for acute myocardial infarction (AMI), stroke, and heart failure in the Tuscany region of Italy.

**Methods:** We carried out a population-based controlled interrupted time series study using segmented linear regression with an autoregressive error term based on admissions data from all public hospitals in Tuscany. The primary outcome measure was weekly hospitalization rates; secondary outcomes included length of stay, and in-hospital mortality.

**Results:** The implementation of the pandemic-related mitigation measures and fear of infection was associated with large decreases in inpatient hospitalization rates overall (−182 [−234, −130]), unplanned hospitalization (−39 [−51, −26]), and for AMI (−1.32 [−1.98, −0.66]), stroke (−1.51 [−2.56, −0.44]), and heart failure (−8.7 [−11.1, −6.3]). Average length of stay and percent in-hospital mortality for select acute medical conditions did not change significantly.

**Conclusion:** In Tuscany, Italy, the COVID-19 pandemic was associated with large reductions in hospitalization rates overall, as well as for heart failure, and the time sensitive conditions of AMI and stroke during the months January to July 2020.

## Introduction

The spread of severe acute respiratory syndrome coronavirus 2 worldwide caused an acute respiratory disease—coronavirus 2019 (COVID-19) pandemic, affecting health and livelihoods worldwide. Italy was severely affected during the months following February 2020 [1]. To limit the spread of SARS-CoV-2 virus and prevent health system collapse, the Italian government responded with a series of regional and national measures that were only gradually lifted in May 2020 [2] (Supplementary Material S1). These measures included the closure of all non-essential businesses, travel restrictions, respiratory hygiene rules, and freedom of movement restrictions [3]. While evidence shows the effectiveness of these interventions at reducing viral transmission [4–6], their unintended impact on healthcare utilization for non-COVID patients is also gaining attention [7, 8].

Decreased healthcare utilization during epidemics is expected due to supply-side factors such as large-scale reorganization of services including postponing non-urgent care to accommodate for a surge of pandemic-affected patients. From the demand-side, care may be missed due to fear of infection for those seeking care or discouragement to access care due to regional lockdown guidelines [9]. Emerging evidence in Italy suggested decreased healthcare utilization among patients with time-sensitive and potentially life-threatening medical conditions [10–12]. However, these studies are limited by simplistic study designs comparing single pre-post time points that do not account for external confounders such as underlying trends [10–13]. We aim to quantify the scale of healthcare utilization changes in unplanned non-COVID related care, and specifically for AMI, stroke, and heart failure in the Italian region of Tuscany during the COVID-19 pandemic using a controlled interrupted time series (cITS) design. Our results may inform healthcare resource planning during pandemics and for the longer-term impact of delayed treatment of acute illnesses.

While randomized controlled trials are considered the gold standard for evaluating intervention impact, randomization is oftentimes impractical, unethical, or economically infeasible [14]. Advances in quasi-experimental designs outlines a range of approaches to improve causal inference when evaluating population-level interventions when randomization is infeasible [15–17]. No regions in Italy were left unaffected by the COVID-19 pandemic and containment measures were implemented nation-wide. Thus, there exists no distinct exposed and unexposed regions to undertake methods such as difference-in-difference analysis, propensity score matching, or synthetic control [18]. Interrupted time series analysis is a robust quasi-experimental design in which comparisons are made within a population across time whereby two groups are separated in time on either side of the interruption [19, 20]. By projecting pre-interruption trend into the post-interruption period as the counterfactual, we effectively control for within-group characteristics over time such as regression to the mean and between group differences such as selection bias and other confounders (e.g., age, sex, and comorbidities etc.). Furthermore, by including a comparable control series, co-interventions apart from underlying trends can be accounted for and thus strengthens causal inference [15, 19].

## Methods

We followed the Reporting of studies conducted using Observational Routinely-collected health Data (RECORD) statement (Supplementary Material S2).

### Study Design and Data Sources

We used cITS to assess the impact of COVID-19 containment interventions on inpatient hospitalizations, average length of hospital stay, and in-hospital mortality following the work of Bernal and colleagues [19]. By analyzing data collected at regular intervals over time, we account for underlying trends when making pre-post comparisons providing more accuracy in our results [19].

We used de-identified inpatient admissions data, inclusive of 41 public hospitals and 10 private facilities across Tuscany, made available quarterly by the Tuscan region. The data were received in September 2020 and analyzed from October 2020 to March 2021. We utilized data from the first week of January to the last week of July during the years 2015–2020. Census data was obtained from the Italian Statistics Bureau, with a projection for 2020 based on previous year trends. The study was carried out in compliance with Italian law on privacy, and approval by an Ethics Committee was not required.

### Setting

Tuscany has 41 public and 10 private hospitals located within 34 health districts serving 3.7 million citizens. During the first wave of COVID-19 (March 2020), Tuscany was moderately affected compared to Lombardy and Veneto, but had also increased the number of ICU beds to meet the needs of increased demand due to COVID-19 patients. Additionally, in April 2020, telehealth services were activated following a regional act to support the ongoing management and care of non-COVID-19 patients.

### Analytic Sample and Variables of Interest

Our primary outcome of interest was the weekly number of patients in the hospital per 100,000 population, overall and for specific conditions of AMI, stroke, and heart failure. Our secondary outcomes were the weekly average length of stay, and percent in-hospital mortality, the latter defined as the percentage of existing hospitalizations records with a discharge code of “deceased” out of all hospitalizations per week. While average length of stay is typically used as an indicator of efficiency, we used it as a proxy for organizational changes in healthcare resource utilization. We hypothesized that average length of stay for patients without COVID-19 decreased due to diversion of healthcare resources towards COVID-19 care. To better understand illness severity, we examined the percent of in-hospital mortality. We hypothesized that the percent of in-hospital mortality for patients without COVID-19 increased due to less severely ill patients disproportionately avoiding hospital care during the pandemic.

Our analytic sample consisted of all inpatient hospitalizations between the months of January to July, for the years 2015–2020, excluding COVID-hospitalizations using the International Classification of Diseases, Ninth Revision, Clinical Modification (*ICD-9*, details in Supplementary Material S3). De-identified data were retrospectively retrieved from the inpatient administrative databases, and patients with AMI, stroke and heart failure were identified using ICD-9 codes (Supplementary Material S3). Inpatient hospitalization was categorized as unplanned, planned, or other. Unplanned care hospitalizations consisted of admissions from the emergency department or direct admissions *via* ambulance arrival. Planned hospitalizations included all pre-scheduled hospitalizations. Other admissions include birthing and psychiatric hospitalizations.

### Analysis

While not required in interrupted time series analysis, we added a control group to account for seasonal effects and thus strengthen causal inference of our results. We selected a historical control as the pandemic’s far reaching impact did not allow for another suitable control group [19]. In this cITS, we defined the exposure group as year 2020 and control as the average of 2015–2019. Historical cohort controls have been previously used to evaluate the impact of a drug funding restriction policy on outcomes such as drug expenditure, primary care visits, and admissions to emergency departments [21]. Our unit of observation was at the patient hospitalization level and our unit of analysis was aggregated at the weekly level. We defined the “interruption” as the combined effects of the COVID containment measures and fear of infection since it is impossible to disentangle the specific contributions of each. We defined week 10 of 2020 as the time of “interruption”; that is when the Italian government issued a nationwide lockdown. We split the data into three separate time periods—pre-lockdown, phase-in, and post-lockdown (Table 1). With the first COVID-death registered on 22nd February 2020 (week 8) and the announcement of a nationwide lockdown on 10th March 2020 (week 10), we expected that the combined influence of increased media coverage and local measures to affect healthcare seeking behaviour gradually initially, and abruptly following the nation-wide lockdown [22]. Thus, we specified weeks 8–10 as the phase-in period and were excluded from analyses (insufficient time points to specify as a separate time period) [23].

**TABLE 1:** Study Time Period. Changes in acute myocardial infarction, stroke and heart failure hospitalizations during COVID-19 pandemic in Tuscany—an interrupted time series study, Tuscany, Italy, 2020.

Time period		Pre-lockdown	Phase-in period	Post-lockdown
Treatment (2020)	Date	January 4–February 22	February 23–March 9	Mar 10–July 31
	Week	Week 1–Week 7	Week 8 & Week 9	Week 10–Week 30
Control (Average of 2015 to 2019)	Week	Week 1–Week 7	Week 8 & Week 9	Week 10–Week 30

We used segmented linear regression with an autoregressive error model to measure the size of the intervention’s immediate effect (level change), effect on changes in the slope (trend change), and an estimate of the longer-term effect [23–26] (for further details see Supplementary Material S4). Autocorrelation was assessed using Durbin-Watson tests, and the autocorrelation function and partial autocorrelation function were used to identify lag order for model correction. We fit a single interrupted time series as an ordinary least squares model and used autocorrelation function and partial autocorrelation function to identify the lag order and test for autocorrelation in the model error distribution. We then fitted a cITS, model specified indicated here:

$$y=\beta_{0}+\beta_{1}\cdot week+\beta_{2}\cdot \exp osed+\beta_{3}\cdot week\cdot \exp osed+\beta_{4}\cdot post+\beta_{5}\cdot week\cdot post+\beta_{6}\cdot post\cdot \exp osed+\beta_{7}\cdot week\cdot \exp osed\cdot post+\varepsilon$$

To ensure that the parallel trend assumption for cITS hold and that comparability between treatment (2020) and control (average of 2015–2019) on pre-intervention covariates exist, we confirmed that the pre-intervention trend differences (β_3_) were not significant (*p* > 0.05) [27]. In models where the pre-intervention trend differed between treatment and control, we opted for the most recent year where the trend difference was not significant to serve as control. We report the level change, trend change, and a long-term estimate of the interruption effect for each interrupted time series model. Level change (β_6_) is the difference in mean scores before and after the interruption and represents the size of the interruption’s immediate impact. To facilitate easier interpretation, we also report level change in relative terms (termed relative change thereafter) calculated as the percentage change relative to the counterfactual. Trend change (β_7_) represents the change in slope gradient following the interruption and quantifies the interruption effect on the overall mean. And lastly, we report the interruption’s estimated longer-term effect in the last week of August using the projected level changed based on the modeled counterfactual trend extended into the post-interruption period.

We conducted subgroup analyses by stratifying the population by sex (male and female), and age groups (under 60, 60 to 69, 70 to 79, 80 to 85, and over 86).

All analyses were conducted in R 4.0.3. All *p*-values are 2-sided and a value of *p* < 0.05 was considered statistically significant.

## Results

Full regression results for both single and controlled interrupted time series are included in Supplemenary Material S5. Graphs depicting the level and trend changes for condition-specific interrupted time series are in Supplemenary Material S6. Subgroup analyses results are in Supplementary Material S7.

### Inpatient Hospitalizations

**FIGURE 1:**
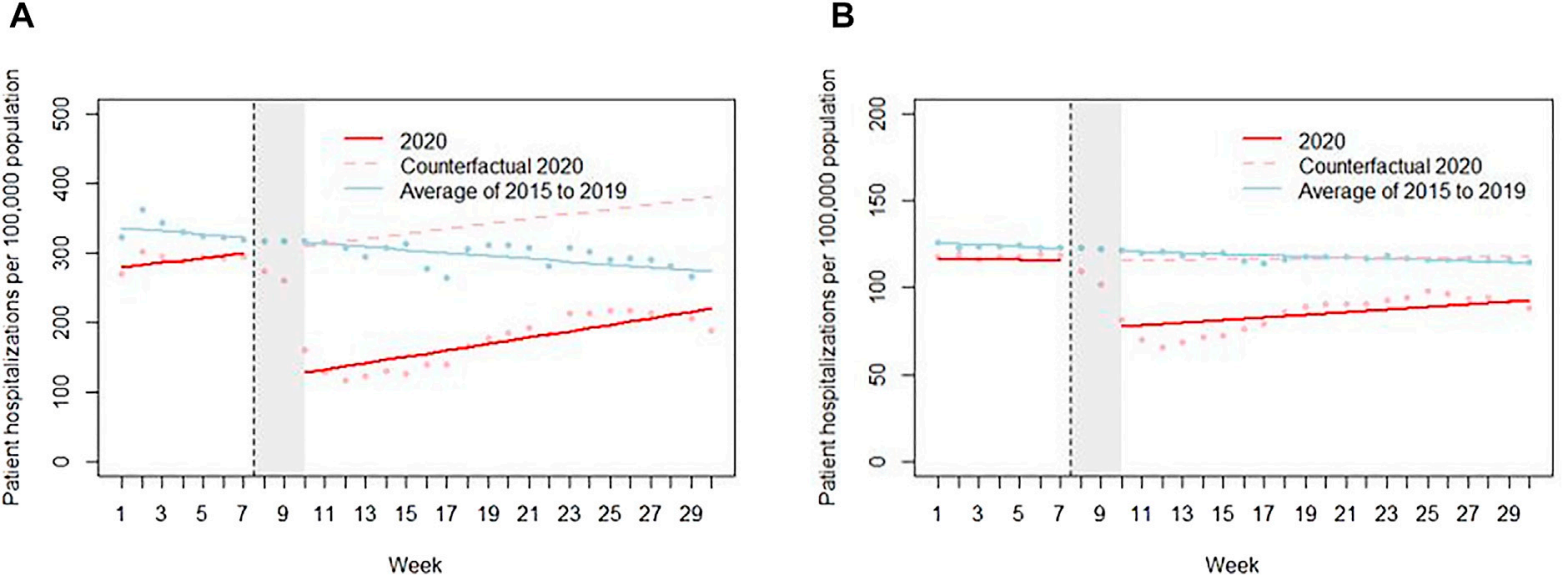
Weekly hospitalization rates per 100,000 population in 2020 compared to corresponding weekly average of previous years (2015–2019) in **(A)** All inpatient care **(B)** Unplanned care. Points represent the raw data; solid lines represent the fitted line; and the dotted line represents the counterfactual. Changes in acute myocardial infarction, stroke and heart failure hospitalizations during COVID-19 pandemic in Tuscany—an interrupted time series study, Tuscany, Italy, 2020.

There were 255,882 non-COVID inpatient hospitalizations involving 206,115 patients (54% female; mean age of 57.2 ± 26.9 years) during January-July 2020 (Table 2). The inpatient hospitalization rate decreased by 182/100,000 (95%CI: -235, -130) following the interruption, representing a 56% decrease (Figure 1A; Table 3). This level change was followed by an insignificant trend increase of 1.04 per 100,000 population per week (95% CI: −8.16, 10.24).

**TABLE 2:** Patient demographic for inpatient care, urgent care, acumte myocardial infarction (AMI), stroke, and heart failure hospitalizations from January to July, 2015 to 2020 in Tuscany (excluding COVID admissions. Changes in acute myocardial infarction, stroke, and heart failure hospitalizations during COVID-19 pandemic in Tuscany - an interrupted time series study, Tuscany, Italy, 2020.

	2015	2016	2017	2018	2019	2020
Inpatient Care						
N	304156	293728	293311	289771	285618	206115
Sex, female, n (%)	164012 (54%)	158257 (54%)	157258 (54%)	155216 (54%)	151525 (54%)	110529 (54%)
Age, mean years (SD)	54.8 (27.1)	55.0 (27.1)	55.3 (27.2)	55.7 (27.1)	56.5 (27.0)	57.2 (26.9)
Urgent care						
N	127137	123313	124674	122528	122386	97988
Sex, female, n (%)	71453 (56)	69309 (56)	70036 (56)	68770 (56)	68023 (56)	54950 (56)
Age, mean years (SD)	63.7 (25.3)	63.6 (25.2)	63.9 (25.0)	64.3 (24.9)	64.9 (24.8)	64.8 (24.5)
AMI						
N	5347	5315	4965	4913	5157	3974
Sex, female, n (%)	2013 [38]	1840 [35]	1711 [35]	1775 [36]	1825 [36]	1333 [34]
Age, mean years (SD)	73.4 (13.4)	72.8 (13.2)	73.3 (12.9)	73.5 (13.1)	72.8 (13.1)	72.7 (13.0)
Stroke						
N	6632	6476	6336	6267	6318	5317
Sex, female, n (%)	3262 (49)	3208 (50)	3146 (50)	3114 (48)	3004 (48)	2602 (49)
Age, mean years (SD)	76.5 (13.7)	76.6 (13.6)	76.2 (14.2)	76.5 (14.0)	76.2 (14.0)	76.3 (13.9)
Heart Failure						
N	15184	14728	15192	15038	15825	12263
Sex, female, n (%)	7457 (49)	7154 (49)	7328 (48)	7420 (49)	7783 (49)	5946 (49)
Age, mean years (SD)	80.7 (11.5)	80.5 (11.7)	80.8 (11.4)	81 (11.4)	81.1 (11.6)	81.2 (11.3)

**TABLE 3:** Interrupted time series results for change in outcome after COVID-19 containment measures. Segmented regression model parameter estimates, 95% CI and *p*-value for (a) weekly hospitalization rates (b) mean length of stay and (c) weekly percent discharged home in Tuscany between January and July. Changes in acute myocardial infarction, stroke and heart failure hospitalizations during COVID-19 pandemic in Tuscany—an interrupted time series study, Tuscany, Italy, 2020.

	Level change			Trend change		
	Effect	95% CI	*p-*value	Effect	95% CI	*p*-value
Hospitalization rate (per 100000)						
All Inpatient	−182	(−235, −130)	<0.001	1.04	(−8.16, 10.24)	0.826
Urgent Care	−39	(−51.1, −26.0)	<0.001	0.67	(−1.99, 3.34)	0.624
AMI	−1.31	(−1.98, −0.66)	<0.001	−0.04	(−0.20, 0.12)	0.635
Stroke	−1.51	(−2.57, −0.44)	0.008	0.05	(−0.13, 0.24)	0.564
Heart Failure	−8.71	(−11.12, −6.29)	<0.001	0.33	(−0.09, 0.75)	0.129
Average length of stay (days)						
AMI	0.63	(−0.31, 1.57)	0.183	0.07	(−0.11, 0.25)	0.427
Stroke	0.82	(−0.95, 2.58)	0.355	−0.01	(−0.32, 0.29)	0.924
Heart Failure	0.01	(−0.69, 0.71)	0.981	−0.06	(−0.19, 0.06)	0.290
In-hospital mortality (%)						
AMI	−1.39	(−4.73, 1.94)	0.406	−0.17	(−0.75, 0.40)	0.548
Stroke	0.27	(−1.45, 1.98)	0.763	0.04	(−0.28, 0.37)	0.794
Heart Failure	2.43	(−0.73, 5.60)	0.138	−0.36	(−0.91, 0.19)	0.207

Subgroup analyses showed no significant differences in inpatient hospitalization rates between male (−88, 95% CI [−116, −60]) and female (−89, 95% CI [−117, −61]). We did find decreased hospitalization rates of patients under the age of 60 (−87, 95% CI [−115, −59]) compared to other age groups (Supplementary Material S7A).

### Unplanned Care Hospitalizations

Unplanned hospitalizations represented 46.5% of all non-COVID inpatient hospitalizations, which is higher than the average of 40% seen in previous years. Unplanned hospitalization remained relatively stable at 116 per 100,000 population prior to the interruption. This was followed by an immediate decrease of 39/100,000 (95% CI: −51.1, −26.0) hospitalizations, representing a 32% decrease (Figure 1B; Table 3). A non-significant positive trend of 0.65 per 100,000 population per week (95% CI: −1.99, 3.34) was observed post interruption, with a rate of 92/100,000 population in the last week of July.

Subgroup analyses show no significant differences in unplanned care hospitalization rates between male (−21, 95% CI [−27, −14]) and female (−22, 95% CI [−29, −15]). Hospitalization rates decrease was larger for those under the age of 60 (−15, 95% CI [−20, −9]) compared to other age groups (Supplementary Material S7B).

### AMI Hospitalizations

In 2020, 1,333 patients (34% female; mean age of 72.7 ± 13.0 years) were hospitalized for AMI at a markedly lower rate than in previous years (4.06 compared to 5.40/100,000, Supplementary Material S6A). Following the interruption, there was a level change of −1.32/100,000 (95% CI: −1.98, −0.66) in AMI hospitalizations, representing a 30% decrease compared to counterfactual (Table 3). This was followed by an insignificant trend change of (−0.04, 95% CI: −0.2, 0.1). By the last week of July 2020, AMI hospitalizations had recovered somewhat to 3.36/100,000. Average length of stay for AMI hospitalizations was stable pre-interruption with no significant level change following interruption (0.6 days, 95% CI: −0.31, 1.57, Supplementary Material S6B). There was no significant in-hospital mortality changes for AMI hospitalizations (−1.39, 95% CI: −4.73, 1.94).

Subgroup analyses show no significant differences in hospitalization rates between male (−0.84, 95% CI [−1.62, −0.06]) and female (−0.55, 95% CI [−0.80, −0.31]) (Supplementary Material S7C). There were no obvious differences in hospitalization rates between age groups. There was a non-significant larger decrease in hospitalization for NSTEMI (−0.84, 95% CI: −1.39, −0.28; 32% reduction compared to counterfactual) than for STEMI (−0.59, 95% CI: −1.06, −0.11; 12.6% reduction compared to counterfactual; Supplementary Material S7C).

### Stroke Hospitalizations

In 2020, 5,317 patients (49% female; mean age of 76.3 ± 13.7 years) were hospitalized for stroke, which is comparable to previous years (6.30 compared to 6.23/100,000, Supplementary Material S6D). Following the interruption, a level change of -1.51/100,000 hospitalizations (95% CI: −2.57, −0.44) occurred, representing a 23% decrease in hospitalizations, with no significant trend change (0.05/100,000, 95%CI: −0.13, 0.24) (Table 3). By the last of July 2020, stroke hospitalization rate was at 4.92/100,000. The average length of stay remained quite stable during the months between January and July in 2020 with a small, insignificant increase of 0.82 days (95% CI: −0.94, 2.58) after the interruption. In-hospital mortality rate for stroke hospitalizations was lower in the months of January and February of 2020 compared to the average of previous years. We observed an increase of 5% (95% CI: −0.27%, 10.82%) after the interruption, bringing the in-hospital mortality rate to 14%, which was comparable to previous year.

Subgroup analyses show no significant differences in hospitalization rates between male (−0.86, 95% CI [−1.45, −0.27]) and female (−0.75, 95% CI [−1.41, −0.09]) (Supplementary Material S7D). We did not observe significant differences in hospitalization rates between patients admitted with ischemic (−0.92, 95% CI [−1.60, −0.26]) and hemorrhagic stroke (−0.69, 95% CI [−1.26, −0.11]). We did find seniors aged 80 to 85 and 86 years and over to have a larger reduction in hospitalization compared to the younger age groups (80–85: −0.9, 95% CI (−1.2, −0.5); over 86: −0.7, 95% CI (−1.2, −0.3)).

### Heart Failure Hospitalizations

In 2020, 12,263 patients (49% female; mean age 81.2 ± 11.3 years) were hospitalized for heart failure, which was comparable to previous years (17.45 compared to 18.20/100,000, Supplementary Material S6G). Following the interruption, there was a level change of −8.71/100,000 hospitalization (95% CI: −11,13, −6.29), representing a 45% reduction compared to counterfactual (Table 3). The impact of this level change was sustained, with no significant trend changes (0.33/100,000 hospitalizations, 95% CI: −0.09, 0.75). The average length of stay remained quite stable between the months January to July in 2020 (level change: 0.01 days, 95% CI (−0.69, 0.71); trend change: −0.06, 95% CI (−0.19, 0.06)). Similarly, no significant changes were observed in percentage of in-hospital mortality during the study period (level change: 2.43%, 95% CI (−0.73, 5.60); trend change: −0.36, 95% CI (−0.91, 0.19), Table 3).

Subgroup analyses show no significant differences in hospitalization rates between male (−4.55, 95% CI [−5.96, −3.14]) and female (−4.65, 95% CI [−6.14, −3.16]) (Supplementary Material S7E). We observed a larger decrease in hospitalization among seniors over the age of 86 (−3.11, 95% CI [−3.96, −2.26]) compared to other younger age groups.

## Discussion

In this study to quantify changes in hospitalizations following implementation of COVID containment measures in Tuscany, Italy during the early months of the COVID-19 pandemic, we found substantial decreases in hospitalization rates, overall as well as for unplanned hospitalizations, for heart failure, and for the time-sensitive conditions of acute myocardial infarction, and stroke. The initial reductions were followed by a slow trend of increases; however, they had not returned to pre-pandemic levels by the end of July 2020. Notably, Tuscany was moderately affected by COVID-19 compared to other regions in Northern Italy [28], and did not experience overcrowding of hospitals seen in other Italian regions, which may explain the overall reduction in hospitalizations. We did not find significant changes in average length of stay nor percent in-hospital mortality for the three specific conditions studied. Emerging studies have highlighted the inequitable access to care [29–31]; we did not find gender-based differences in hospitalization rate changes. In subgroup analyses, we found a larger decrease in inpatient and unplanned care hospitalization rates among those under the age of 60 compared to the older age groups. Our findings are similar to reports on reduced hospitalization rates observed in countries [32–34]; a systematic review evaluating 81 studies from 20 countries found a median reduction of 28% in hospital admissions during the first wave of the pandemic up to May 2020 [8].

A strength of our analysis was using a cITS design, a robust quasi-experimental design when randomization is infeasible, allowing us to assess the impact of COVID-19 while controlling for pre-existing trends. Recent research highlights a debate of relative strengths, weaknesses, and disciplinary preferences of using cITS and difference-in-difference approaches which largely stems from the variety of ways in which the two methods are defined (see Fry and Hatfield’s review) [35–37]. Guided by our research question and the data structure available–namely, the aggregate count data available on a weekly basis, substantial data points before and after the interruption, and the lack of concurrent control, we deemed controlled interrupted time series to be the most appropriate and robust analytical approach [15, 19]. Additionally, in the absence of available concurrent control, using a historical control allowed us to ensure no spillover and pre-implementation fit due to the demographic similarities.

While identifying the specific mechanisms leading to this substantial decline in hospitalization is outside the scope of this study, we propose that the observed reduction is likely multifactorial. Factors may include patients’ fear of contracting infection in hospitals, cancelling of non-urgent procedures and treatments, and potential excess hospitalization pre-pandemic. Results from our study showed that reduction in hospitalization rates had occurred prior to the nation-wide lockdown, and that the rates had not rebounded after the lockdown lifted, which may suggest that patient’s fear of contagion may be more plausible. A recent survey found that between 20.1% of the sampled participants in Tuscany (*n* = 648) indicated that they had forgone healthcare services despite an indicated need during the pandemic [38]. Correspondingly, D’Ascenzi and colleagues found patient fear to be a main cause for the observed reduction in emergency calls for cardiac related symptoms and subsequent hospital admission in Tuscany between January and March 2020 [39]. Within Tuscany, Italian National Institute of Statistics (ISTAT) reported a 26% increase in mortality at home and 14% increase in mortality in long-term care facilities for cardiovascular diseases when comparing 2015–2019 to 2020 [40]. Strict stay-at-home orders from the Italian government during the lockdown period coupled with media outlets indicating high viral transmission rates in hospitals may have discouraged patients from seeking timely medical attention despite need.

Studies have reported increased mortality rates and complications from AMI and stroke during the pandemic compared to previous years [13, 41, 42]. Thus, we expected an increase in percentage of in-hospital mortality rates for non-COVID urgent conditions such as AMI and stroke. However, we did not find evidence of significant changes in percentage of in-hospital mortality and average length of stay after the implementation of COVID-containment measures as compared to prior weeks when controlling for underlying trends. This finding differs from reports of increased in-hospital mortality from Northern Italy, which was severely affected by COVID-19 pandemic in the first wave [10, 43, 44].

Strategies implemented in Tuscany to cope with the expected surge of COVID-related health service use and prevent overcrowding of emergency rooms may also have contributed to the maintenance of optimal care for non-COVID patients as suggested by the absence of significant changes in average length of stay and in-hospital mortality. In Tuscany, hospitals were reorganized during the pandemic to operate either as a dual-track system or COVID-only, with dual-track systems [45] clearly separating services for COVID-19 patients from other essential health services. In a quality-of-care monitoring network set up at the beginning of the COVID-19 pandemic (Mimico-19), Spadea and colleagues found that regardless of COVID-19 burden on the seven Italian regions under investigation, timely and effective response was maintained for time-dependent care pathways in hospital across the clinical areas—cardiology, oncology, and orthopaedics, with the exception of treatment of patients presenting with STEMI in Lombardia [46].

Among the condition-specific analyses, we found a much larger percentage reduction in hospitalization rate for heart failure compared to AMI and stroke. This may be because many, if not most, patients hospitalized for heart failure are mainly managed by titrations of diuretics and antihypertensives, which may have more flexibility and potential at being accomplished in the outpatient setting than previously accepted [47]. Heart failure is the second leading cause of hospitalization in Italy [48] and is associated with high expenditures and frequent rehospitalizations [49]. The patterns of care during the pandemic may hold lessons for more cost-effective heart failure care, such as implementing validated risk stratification tools for admission decisions to prevent unnecessary hospitalization [50]. Utilizing telemedicine has shown to be feasible in stabilizing heart failure symptoms and reducing readmission rates in an Italian pilot study [51]. In fact, a regional act was passed in Tuscany in April 2020 to promote the use of telemedicine in maintaining a standard of care for patients with chronic illness such as heart failure to alleviate hospitalization.

While substantial overall excess mortality rate was observed during the COVID-19 pandemic, Gianicolo and colleagues et al found that sex- and age-adjusted excess mortality was not substantially different from the official number of registered COVID-19 deaths between February and June in 2020 in Italy [52], suggesting there were no significant excess mortality from non-COVID causes. While we did not find evidence of increased in-hospital mortality across the three condition, future analyses on other indicators such as out-of-hospital mortality rates could provide us with a fuller picture of the extent of impact on the overall mortality rates. Additionally, while delaying care may not impact survival in the short term, there remain concerns that the delayed treatments of urgent conditions will translate to subsequent increase in hospitalization, disability, and mortality [53–55]. For example, among patients presenting with AMI, early restoration of blood flow is associated with lower rates in mortality (among high-risk patients), reinfarction rates, and stroke incidence [54]. As well, changes in stroke management to prioritize COVID-19 patient care exposes patients to increased risk of complications and recurrent events [56].

### Limitations

Our study has important limitations. First, due to the rapidly evolving and unpredictable nature of the pandemic, we were unable to pinpoint a specific time point when the interruption took effect. Additionally, we were unable to differentiate between more direct impacts of the pandemic and the associated containment measures. To account for both, we had specified a two-week phase-in period to such that both events to take effect. Second, we did not have access to mortality data outside of the hospital, and thus were unable to determine the full extent of impact in delayed care for time-sensitive conditions. Studies from Emilia-Romagna [57] and Lombardia [43] have found an increase in out-of-hospital mortality due to cardiac causes in the first wave of the pandemic. While Tuscany was relatively less affected by COVID-19 compared to other regions in Italy, complementary studies that examine cardiac conditions care pathway process measures such as door-to-balloon time [58] can better elucidate interpretations. For example, Rossi and colleagues found that in-hospital mortality rates for AMI patients did not change significantly after COVID-19 in Brescia despite an increase in door-to-balloon time, pointing towards a threshold of care management of keeping this measure to 95 min for AMI patients [59]. Third, we were limited to the data available and utilized the percentage of hospital mortality and average length of stay to serve as indicators for quality-of-care management. Future studies may consider collecting data that support measures such as 30-day readmission rates and post-discharge mortality rates to further enhance interpretations of quality-of-care management in hospital.

### Conclusion

The COVID-19 pandemic was associated with substantial reductions in hospitalization rates in Tuscany, Italy, but was not associated with changes in hospital length of stay and percentage in-hospital mortality for patients hospitalized for AMI, stroke, and heart failure. The full impact of delayed treatment for these time-sensitive acute conditions remains unclear.
