# Supplementary material for: Changes in Acute Myocardial Infarction, Stroke, and Heart Failure Hospitalizations During COVID-19 Pandemic in Tuscany—An Interrupted Time Series Study
Source: Int J Public Health. 2022 Jun 8;67:1604319. doi: 10.3389/ijph.2022.1604319 (PMC9216172; doi:10.3389/ijph.2022.1604319)
Supplement: Supplementary file 1 [file DataSheet1.docx]

**Title:** Changes in acute myocardial infarction, stroke and heart failure hospitalizations during COVID-19 pandemic in Tuscany – an interrupted time series study

**Journal name:** International Journal of Public Health

**Supplement**

**S1 -**Timeline of major COVID-19 events in Italy. **Changes in acute myocardial infarction, stroke and heart failure hospitalizations during COVID-19 pandemic in Tuscany – an interrupted time series study, Tuscany, Italy, 2020.**

| **Date** | **Week** | **Description** |
| --- | --- | --- |
| January 31^st^, 2020 | Week 4 | First 2 cases confirmed in Rome |
| February 22^nd^, 2020 | Week 8 | First death confirmed  79 cases confirmed |
| March 8^th,^ 2020 | Week 10 | Lombardy and 14 states in lockdown  7375 cases confirmed |
| March 10^th,^ 2020 | Week 10 | Nation-wide lockdown (all non-essential movement); rules were unclear, enforcement was spotty  10148 cases confirmed |
| March 11^th,^ 2020 | Week 10 | All restaurants and bars closed |
| March 22^nd^, 2020 | Week 12 | Factories closed and nonessential production halted; Outdoor activities banned, parks were closed. |
| May 3^rd^,2020 | Week 18 | Freedom of movement within municipality re-instated |
| May 18^th^, 2020 | Week 20 | Freedom of movement within region re-instated |
| June 3^rd^, 2020 | Week 22 | Freedom of movement in Italy reinstated |
| June 15^th^, 2020 | Week 24 | Theaters, swimming pools, and gyms reopened |

**S2 – Strobe Checklist**

STROBE Statement—checklist of items that should be included in reports of observational studies. **Changes in acute myocardial infarction, stroke and heart failure hospitalizations during COVID-19 pandemic in Tuscany – an interrupted time series study, Tuscany, Italy, 2020.**

|  | Item No | Recommendation | Page  No |
| --- | --- | --- | --- |
| **Title and abstract** | 1 | (*a*) Indicate the study’s design with a commonly used term in the title or the abstract | 1 |
|  |  | (*b*) Provide in the abstract an informative and balanced summary of what was done and what was found | 1 |
| Introduction | | | |
| Background/rationale | 2 | Explain the scientific background and rationale for the investigation being reported | 2 |
| Objectives | 3 | State specific objectives, including any prespecified hypotheses | 2 |
| Methods | | | |
| Study design | 4 | Present key elements of study design early in the paper | 2-3 |
| Setting | 5 | Describe the setting, locations, and relevant dates, including periods of recruitment, exposure, follow-up, and data collection | 3 |
| Participants | 6 | (*a*) *Cohort study*—Give the eligibility criteria, and the sources and methods of selection of participants. Describe methods of follow-up  *Case-control study*—Give the eligibility criteria, and the sources and methods of case ascertainment and control selection. Give the rationale for the choice of cases and controls  *Cross-sectional study*—Give the eligibility criteria, and the sources and methods of selection of participants |  |
|  |  | (*b*) *Cohort study*—For matched studies, give matching criteria and number of exposed and unexposed  *Case-control study*—For matched studies, give matching criteria and the number of controls per case |  |
| Variables | 7 | Clearly define all outcomes, exposures, predictors, potential confounders, and effect modifiers. Give diagnostic criteria, if applicable | 3-4 |
| Data sources/ measurement | 8* | For each variable of interest, give sources of data and details of methods of assessment (measurement). Describe comparability of assessment methods if there is more than one group | 3-4 |
| Bias | 9 | Describe any efforts to address potential sources of bias | 4 |
| Study size | 10 | Explain how the study size was arrived at | n/a |
| Quantitative variables | 11 | Explain how quantitative variables were handled in the analyses. If applicable, describe which groupings were chosen and why | 4 |
| Statistical methods | 12 | (*a*) Describe all statistical methods, including those used to control for confounding | 4-5 |
|  |  | (*b*) Describe any methods used to examine subgroups and interactions | 5 |
|  |  | (*c*) Explain how missing data were addressed | 5 |
|  |  | (*d*) *Cohort study*—If applicable, explain how loss to follow-up was addressed  *Case-control study*—If applicable, explain how matching of cases and controls was addressed  *Cross-sectional study*—If applicable, describe analytical methods taking account of sampling strategy | n/a |
|  |  | (*e*) Describe any sensitivity analyses | n/a |

| Results | | | |
| --- | --- | --- | --- |
| Participants | 13* | (a) Report numbers of individuals at each stage of study—eg numbers potentially eligible, examined for eligibility, confirmed eligible, included in the study, completing follow-up, and analysed | 6-8 |
|  |  | (b) Give reasons for non-participation at each stage | n/a |
|  |  | (c) Consider use of a flow diagram | n/a |
| Descriptive data | 14* | (a) Give characteristics of study participants (eg demographic, clinical, social) and information on exposures and potential confounders | 5-7 |
|  |  | (b) Indicate number of participants with missing data for each variable of interest | 5-7 |
|  |  | (c) *Cohort study*—Summarise follow-up time (eg, average and total amount) | n/a |
| Outcome data | 15* | *Cohort study*—Report numbers of outcome events or summary measures over time | n/a |
|  |  | *Case-control study—*Report numbers in each exposure category, or summary measures of exposure | n/a |
|  |  | *Cross-sectional study—*Report numbers of outcome events or summary measures | n/a |
| Main results | 16 | (*a*) Give unadjusted estimates and, if applicable, confounder-adjusted estimates and their precision (eg, 95% confidence interval). Make clear which confounders were adjusted for and why they were included | 5-8 |
|  |  | (*b*) Report category boundaries when continuous variables were categorized | n/a |
|  |  | (*c*) If relevant, consider translating estimates of relative risk into absolute risk for a meaningful time period | n/a |
| Other analyses | 17 | Report other analyses done—eg analyses of subgroups and interactions, and sensitivity analyses | 5-8 |
| Discussion | | | |
| Key results | 18 | Summarise key results with reference to study objectives | 8-9 |
| Limitations | 19 | Discuss limitations of the study, taking into account sources of potential bias or imprecision. Discuss both direction and magnitude of any potential bias | 11-12 |
| Interpretation | 20 | Give a cautious overall interpretation of results considering objectives, limitations, multiplicity of analyses, results from similar studies, and other relevant evidence | 9-11 |
| Generalisability | 21 | Discuss the generalisability (external validity) of the study results | n/a |
| Other information | | | |
| Funding | 22 | Give the source of funding and the role of the funders for the present study and, if applicable, for the original study on which the present article is based | N/a |

*Give information separately for cases and controls in case-control studies and, if applicable, for exposed and unexposed groups in cohort and cross-sectional studies.

**Note:** An Explanation and Elaboration article discusses each checklist item and gives methodological background and published examples of transparent reporting. The STROBE checklist is best used in conjunction with this article (freely available on the Web sites of PLoS Medicine at http://www.plosmedicine.org/, Annals of Internal Medicine at http://www.annals.org/, and Epidemiology at http://www.epidem.com/). Information on the STROBE Initiative is available at www.strobe-statement.org.

**S3** – ICD 9 codes for acute myocardial infarction, stroke and heart failure. **Changes in acute myocardial infarction, stroke and heart failure hospitalizations during COVID-19 pandemic in Tuscany – an interrupted time series study, Tuscany, Italy, 2020.**

*COVID-19*

"07889", "4848", "4660", "490", "5198", "51882".

*AMI* "41090","41091","41092","41070","41071","41072","41001","41002","41010","41011","41012","41020","41021","41022","41030","41031","41032","41040","41041","41042","41050","41051","41052","41060","41061","41062","41080","41081","41082

*Stroke*

"43301","43311","43321","43331","43381","43391","43401","43411","43491","430","431","432","4320","4321","4329"

*Heart failure*

"4280","4281","42820","42821","42822","42823","42830","42831","42832","42833","42840","42841","42842","42843","4289"

**S4–** Segmented regression model specification. **Changes in acute myocardial infarction, stroke and heart failure hospitalizations during COVID-19 pandemic in Tuscany – an interrupted time series study, Tuscany, Italy, 2020.**

The model specified for this analysis is as follows:

$$Y= \beta0+\beta1 week + \beta2 post + \beta3 week\times post + \varepsilon$$

$$Y= \beta0+\beta1 week + \beta2 exposed + \beta3 week\times exposed + \beta4 post + \beta5 week\times post + \beta6 post\times exposed + \beta7 week\times exposed\times post + \varepsilon$$

In this model, week is a discrete variable ranging from 1 to 30, where week 1 represents the first week in January and week 30 represents the final week in July. Exposed and post are both dummy variables, with exposed =1 indicating the group 2020 and 0 indicating the control group; and post =1 indicating after the interruption and post =0 indicating before the interruption.

In regards to the model coefficients, β_0_ represents the baseline level for the control group, β_1_ represents the baseline trend for the control group, β_2_ represents the existing level difference between treatment and control, β_3_ represents existing trend difference between treatment and control, β_4_ represents the difference in baseline level between the treatment and control group, β_5_ represents the slope change following the interruption, β_6_ represents the level change between the intervention and control group associated with the interruption, and β_7_ represents the slope change between the intervention and control group, associated with the interruption.


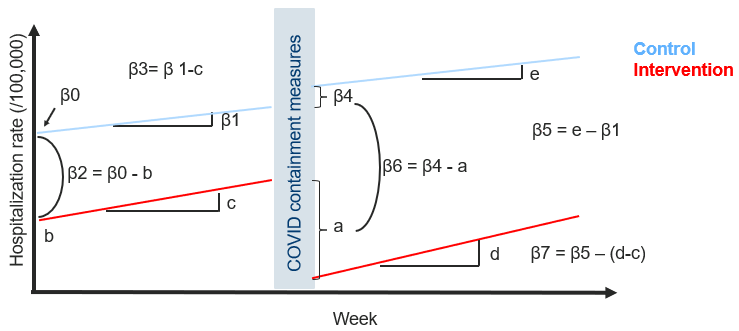


**S5– Interrupted time series model results -** Parameter estimates, P-values, and 95% confidence intervals of the full segmented regression model for weekly hospitalization rates (/100,000), average length of stay, and weekly percent discharged home (%). (a) All inpatient – hospitalization rate (/100,000); (b)Unplanned – Hospitalization rate (/100,000); (c) AMI hospitalizations – Hospitalization rate (/100,000); (d) AMI hospitalizations – Average length of stay (days); (e) AMI hospitalizations – Percent in-hospital mortality (%); (f) Stroke hospitalizations – Hospitalization rate (/100,000); (g) Stroke hospitalizations – Average length of stay (days); (h) Stroke hospitalizations – Percentage in-hospital mortality (%); (i)Heart Failure hospitalizations – Hospitalization rate (/100,000); (j) Heart Failure hospitalizations – Average length of stay (days); (k) Heart Failure hospitalizations – Percentage in-hospital mortality (%). **Changes in acute myocardial infarction, stroke and heart failure hospitalizations during COVID-19 pandemic in Tuscany – an interrupted time series study, Tuscany, Italy, 2020.**

| **Single Interrupted Time Series** | | | | **Controlled Interrupted Time Series** | | | |
| --- | --- | --- | --- | --- | --- | --- | --- |
|  | Coefficient | *p*-value | 95% CI |  | Coefficient | *p*-value | 95% CI |
| 1. All inpatient – Hospitalization rate (/100,000) | | | | | | | |
| $\beta0$ | 276.3 | <0.001 | 242.6, 310.1 | $\beta0$ | 338.8 | <0.001 | 309.6, 368.0 |
| $\beta1$ | 3.2 | 0.4 | -4.2, 10.6 | $\beta1$ | -2.4 | 0.3 | -7.4, 2.5 |
| $\beta2$ | -182.2 | <0.001 | -227.1, -137.2 | $\beta2$ | -62.6 | 0.008 | -107.0, -18.2 |
| $\beta3$ | 1.4 | 0.9 | -6.2, 9.0 | $\beta3$ | 0.1 | 1.0 | -27.1, 27.4 |
|  |  |  |  | $\beta4$ | 0.3 | 0.9 | -5.0, 5.7 |
|  |  |  |  | $\beta5$ | 5.7 | 0.2 | -3.2, 14.5 |
|  |  |  |  | $\beta6$ | -182.3 | <0.001 | -234.5, -130.1 |
|  |  |  |  | $\beta7$ | 1.0 | 0.8 | -8.2, 10.2 |
|  | | | | | | | |
| **Single Interrupted Time Series** | | | | **Controlled Interrupted Time Series** | | | |
|  | Coefficient | *p*-value | 95% CI |  | Coefficient | *p*-value | 95% CI |
| 1. Unplanned – Hospitalization rate (/100,000) | | | | | | | |
| $\beta0$ | 116.3 | <0.001 | 102.1, 130.4 | $\beta0$ | 126.6 | <0.001 | 117.2, 135.9 |
| $\beta1$ | -0.2 | 0.9 | -2.8, 2.4 | $\beta1$ | -0.6 | 0.4 | -2.0, 0.7 |
| $\beta2$ | -36.8 | <0.001 | -52.5, -21.1 | $\beta2$ | -9.8 | 0.2 | -23.4, 3.7 |
| $\beta3$ | 0.9 | 0.6 | -2.0, 3.8 | $\beta3$ | 0.05 | 1.0 | -4.7, 4.8 |
|  |  |  |  | $\beta4$ | 0.3 | 0.7 | -1.4, 2.0 |
|  |  |  |  | $\beta5$ | 0.4 | 0.7 | -1.9, 2.8 |
|  |  |  |  | $\beta6$ | -38.5 | <0.001 | -51.1, -26.0 |
|  |  |  |  | $\beta7$ | 0.7 | 0.6 | -2.0, 3.3 |
|  | | | | | | | |
| **Single Interrupted Time Series** | | | | **Controlled Interrupted Time Series** | | | |
|  | Coefficient | *p*-value | 95% CI |  | Coefficient | *p*-value | 95% CI |
| 1. AMI hospitalizations – Hospitalization rate (/100,000) | | | | | | | |
| $\beta0$ | 4.1 | <0.001 | 3.4, 4.8 | $\beta0$ | 5.4 | 0 | 4.8, 6.0 |
| $\beta1$ | 0.03 | 0.7 | -0.1, 0.2 | $\beta1$ | -0.07 | 0.1 | -0.2, 0.02 |
| $\beta2$ | -1.5 | <0.001 | -2.4, -0.5 | $\beta2$ | -1.3 | 0.003 | -2.2, -0.5 |
| $\beta3$ | 0 | 1.0 | -0.2, 0.2 | $\beta3$ | 0.03 | 0.9 | -0.3, 0.4 |
|  |  |  |  | $\beta4$ | 0.03 | 0.6 | -0.08, 0.1 |
|  |  |  |  | $\beta5$ | 0.1 | 0.2 | -0.04, 0.2 |
|  |  |  |  | $\beta6$ | -1.3 | 0.0003 | -2.0, -0.7 |
|  |  |  |  | $\beta7$ | -0.04 | 0.6 | -0.2, 0.1 |

| 1. AMI hospitalizations – Average length of stay (days) | | | | | | | |
| --- | --- | --- | --- | --- | --- | --- | --- |
| Single Interrupted Time Series | | | | **Controlled Interrupted Time Series** | | | |
|  | Coefficient | *p*-value | 95% CI |  | Coefficient | *p*-value | 95% CI |
| $\beta0$ | 7.5 | <0.001 | 6.8,8.3 | $\beta0$ | 7.2 | <2e-16 | 6.7, 7.7 |
| $\beta1$ | -0.2 | 0.04 | 0.3, -0.008 | $\beta1$ | 0.05 | 0.3 | -0.04, 0.1 |
| $\beta2$ | 0.9 | 0.06 | -0.03,1.8 | $\beta2$ | -0.4 | 0.4 | -1.3, 0.5 |
| $\beta3$ | 0.1 | 0.07 | -0.08, 0.1 | $\beta3$ | -0.2 | 0.3 | -0.7, 0.3 |
|  |  |  |  | $\beta4$ | -0.06 | 0.2 | -0.1, 0.03 |
|  |  |  |  | $\beta5$ | -0.08 | 0.4 | -0.3, 0.1 |
|  |  |  |  | $\beta6$ | 0.6 | 0.2 | -0.3, 1.6 |
|  |  |  |  | $\beta7$ | 0.07 | 0.4 | -0.1, 0.3 |
|  |  |  |  |  |  |  |  |
|  |  |  |  |  |  |  |  |
|  |  |  |  |  |  |  |  |

| 1. AMI hospitalizations – Percent in-hospital mortality (%) | | | | | | | |
| --- | --- | --- | --- | --- | --- | --- | --- |
| **Single Interrupted Time Series** | | | | **Controlled Interrupted Time Series** | | | |
|  | Coefficient | *p*-value | 95% CI |  | Coefficient | *p*-value | 95% CI |
| $\beta0$ | 5.8 | <0.001 | 3.0, 8.6 | $\beta0$ | 7.4 | <0.001 | (5.6,9.2) |
| $\beta1$ | 0.05 | 0.9 | -0.6, 0.7 | $\beta1$ | -0.2 | 0.3 | (-0.5,0.1) |
| $\beta2$ | -1.3 | 0.5 | -5.0, 2.4 | $\beta2$ | -1.6 | 0.3 | (-4.3,1.2) |
| $\beta3$ | -0.009 | 1.0 | -0.6, 0.6 | $\beta3$ | 0.2 | 0.4 | (-0.3,0.8) |
|  |  |  |  | $\beta4$ | 0.1 | 0.9 | (-1.8, 2.0) |
|  |  |  |  | $\beta5$ | 0.2 | 0.3 | (-0.2,0.5) |
|  |  |  |  | $\beta6$ | -1.4 | 0.4 | (-4.7,1.9) |
|  |  |  |  | $\beta7$ | -0.2 | 0.5 | (-0.8,0.4) |
|  |  |  |  |  |  |  |  |
|  |  |  |  |  |  |  |  |
|  |  |  |  |  |  |  |  |

| 1. Stroke hospitalizations – Hospitalization rate (/100,000) | | | | | | | |
| --- | --- | --- | --- | --- | --- | --- | --- |
| **Single Interrupted Time Series** | | | | **Controlled Interrupted Time Series** | | | |
|  | Coefficient | *p*-value | 95% CI |  | Coefficient | *p*-value | 95% CI |
| $\beta0$ | 6.3 | <0.001 | 5.4, 7.1 | $\beta0$ | 6.2 | 0 | 5.6, 6.8 |
| $\beta1$ | -0.03 | 0.8 | -0.2, 0.2 | $\beta1$ | -0.03 | 0.6 | -0.1, 0.08 |
| $\beta2$ | -1.5 | 0.01 | -2.6, -0.5 | $\beta2$ | 0.07 | 0.9 | -0.8, 1.0 |
| $\beta3$ | 0.05 | 0.6 | -0.1, 0.2 | $\beta3$ | 0.1 | 0.7 | -0.5, 0.7 |
|  |  |  |  | $\beta4$ | 0.003 | 1.0 | -0.1, 0.1 |
|  |  |  |  | $\beta5$ | -0.01 | 0.9 | -0.2, 0.2 |
|  |  |  |  | $\beta6$ | -1.5 | 0.008 | -2.6, -0.4 |
|  |  |  |  | $\beta7$ | 0.05 | 0.6 | -0.1, 0.2 |
|  |  |  |  |  |  |  |  |
|  |  |  |  |  |  |  |  |
|  |  |  |  |  |  |  |  |

| 1. Stroke hospitalizations – Average length of stay (days) | | | | | | | |
| --- | --- | --- | --- | --- | --- | --- | --- |
| **Single Interrupted Time Series** | | | | **Controlled Interrupted Time Series** | | | |
|  | Coefficient | *p*-value | 95% CI |  | Coefficient | *p*-value | 95% CI |
| $\beta0$ | 13.0 | <0.001 | 11.5, 14.6 | $\beta0$ | 12.3 | <0.001 | 11.4, 13.3 |
| $\beta1$ | -0.09 | 0.6 | -0.4, 0.3 | $\beta1$ | 0.02 | 0.8 | -0.2, 0.2 |
| $\beta2$ | 1.0 | 0.3 | -1.0, 3.0 | $\beta2$ | 0.7 | 0.3 | -0.7, 2.2 |
| $\beta3$ | -0.06 | 0.7 | -0.4, 0.3 | $\beta3$ | 0.2 | 0.7 | -0.8, 1.2 |
|  |  |  |  | $\beta4$ | -0.04 | 0.6 | -0.2, 0.1 |
|  |  |  |  | $\beta5$ | -0.1 | 0.5 | -0.4, 0.2 |
|  |  |  |  | $\beta6$ | 0.8 | 0.4 | -0.9, 2.6 |
|  |  |  |  | $\beta7$ | -0.01 | <0.001 | -0.3, 0.3 |
|  |  |  |  |  |  |  |  |
|  |  |  |  |  |  |  |  |
|  |  |  |  |  |  |  |  |

| 1. Stroke hospitalizations – Percentage in-hospital mortality (%) | | | | | | | |
| --- | --- | --- | --- | --- | --- | --- | --- |
| **Single Interrupted Time Series** | | | | **Controlled Interrupted Time Series** | | | |
|  | Coefficient | *p*-value | 95% CI |  | Coefficient | *p*-value | 95% CI |
| $\beta0$ | 2.9 | 0.01 | 0.7, 5.2 | $\beta0$ | 3.9 | <0.001 | 3.1, 4.8 |
| $\beta1$ | 0.01 | 1.0 | -0.5, 0.5 | $\beta1$ | 0.03 | 0.7 | -0.1, 0.2 |
| $\beta2$ | -0.2 | 0.9 | -3.2, 2.8 | $\beta2$ | -1.0 | 0.2 | -2.5, 0.5 |
| $\beta3$ | 0.01 | 1.0 | -0.5, 0.5 | $\beta3$ | -0.3 | 0.5 | -1.1, 0.5 |
|  |  |  |  | $\beta4$ | -0.03 | 0.7 | -0.2, 0.1 |
|  |  |  |  | $\beta5$ | -0.04 | 0.8 | -0.4, 0.3 |
|  |  |  |  | $\beta6$ | 0.3 | 0.8 | -1.4, 2.0 |
|  |  |  |  | $\beta7$ | 0.04 | 0.8 | -0.3, 0.4 |
|  |  |  |  |  |  |  |  |
|  |  |  |  |  |  |  |  |
|  |  |  |  |  |  |  |  |

| 1. Heart Failure hospitalizations – Hospitalization rate (/100,000) | | | | | | | |
| --- | --- | --- | --- | --- | --- | --- | --- |
| **Single Interrupted Time Series** | | | | **Controlled Interrupted Time Series** | | | |
|  | Coefficient | *p*-value | 95% CI |  | Coefficient | *p*-value | 95% CI |
| $\beta0$ | 17.4 | <0.001 | 15.2, 19.7 | $\beta0$ | 18.2 | <0.001 | 16.9, 19.5 |
| $\beta1$ | -0.1 | 0.6 | -0.6, 0.4 | $\beta1$ | -0.2 | 0.1 | -0.4, 0.04 |
| $\beta2$ | -7.3 | <0.001 | -10.3, -4.4 | $\beta2$ | -0.7 | 0.5 | -2.8, 1.3 |
| $\beta3$ | 0.2 | 0.4 | -0.3, 0.7 | $\beta3$ | 1.0 | 0.1 | -0.3, 2.4 |
|  |  |  |  | $\beta4$ | -0.1 | 0.4 | -0.4, 0.1 |
|  |  |  |  | $\beta5$ | 0.08 | 0.7 | -0.3, 0.5 |
|  |  |  |  | $\beta6$ | -8.7 | <0.001 | -11.1, -6.3 |
|  |  |  |  | $\beta7$ | 0.3 | 0.1 | -0.09, 0.8 |
|  |  |  |  |  |  |  |  |
|  |  |  |  |  |  |  |  |
|  |  |  |  |  |  |  |  |

| 1. Heart Failure hospitalizations – Average length of stay (days) | | | | | | | |
| --- | --- | --- | --- | --- | --- | --- | --- |
| **Single Interrupted Time Series** | | | | **Controlled Interrupted Time Series** | | | |
|  | Coefficient | *p*-value | 95% CI |  | Coefficient | *p*-value | 95% CI |
| $\beta0$ | 6.3 | <0.001 | 2.1, 4.2 | $\beta0$ |  |  |  |
| $\beta1$ | -0.1 | 0.05 | -0.4, 0.06 | $\beta1$ |  |  |  |
| $\beta2$ | 1.6 | <0.001 | 0.3, 2.8 | $\beta2$ |  |  |  |
| $\beta3$ | 0.03 | 0.6 | -0.09, 0.4 | $\beta3$ |  |  |  |
|  |  |  |  | $\beta4$ |  |  |  |
|  |  |  |  | $\beta5$ |  |  |  |
|  |  |  |  | $\beta6$ |  |  |  |
|  |  |  |  | $\beta7$ |  |  |  |
|  |  |  |  |  |  |  |  |
|  |  |  |  |  |  |  |  |
|  |  |  |  |  |  |  |  |

| 1. Heart Failure hospitalizations – Percentage in-hospital mortality (%) | | | | | | | |
| --- | --- | --- | --- | --- | --- | --- | --- |
| **Single Interrupted Time Series** | | | | **Controlled Interrupted Time Series** | | | |
|  | Coefficient | *p*-value | 95% CI |  | Coefficient | *p*-value | 95% CI |
| $\beta0$ | 9.2 | <0.001 | 6.3, 12.2 | $\beta0$ | 12.1 | 0 | 10.3,13.8 |
| $\beta1$ | 0.2 | 0.6 | -0.5, 0.8 | $\beta1$ | -0.05 | 0.7 | -0.4, 0.2 |
| $\beta2$ | 1.0 | 0.6 | -2.9, 4.8 | $\beta2$ | -2.7 | 0.05 | -5.3, -0.04 |
| $\beta3$ | -0.3 | 0.4 | -0.9, 0.3 | $\beta3$ | -1.3 | 0.2 | -3.1, 0.5 |
|  |  |  |  | $\beta4$ | 0.07 | 0.7 | -0.3, 0.4 |
|  |  |  |  | $\beta5$ | 0.2 | 0.5 | -0.3, 0.7 |
|  |  |  |  | $\beta6$ | 2.4 | 0.1 | -0.7, 5.6 |
|  |  |  |  | $\beta7$ | -0.4 | 0.2 | -0.9, 0.2 |
|  |  |  |  |  |  |  |  |
|  |  |  |  |  |  |  |  |
|  |  |  |  |  |  |  |  |

**S6–** Weekly hospitalization rates per 100,000 population, average length of stay, and percent in-hospital mortality in 2020 compared to corresponding weekly average of previous years (2015 to 2019) in (a)-(c) acute myocardial infarction, (d)-(f) stroke, and (g)-(i) heart failure. Points represent the raw data; solid lines represent the fitted line; and the dotted line represents the counterfactual. **Changes in acute myocardial infarction, stroke and heart failure hospitalizations during COVID-19 pandemic in Tuscany – an interrupted time series study, Tuscany, Italy, 2020.**

| Hospitalizations (/100,000) | Average length of stay (days) | Percent in-hospital mortality (%) |
| --- | --- | --- |
| Acute Myocardial Infarction | | |
| (a)  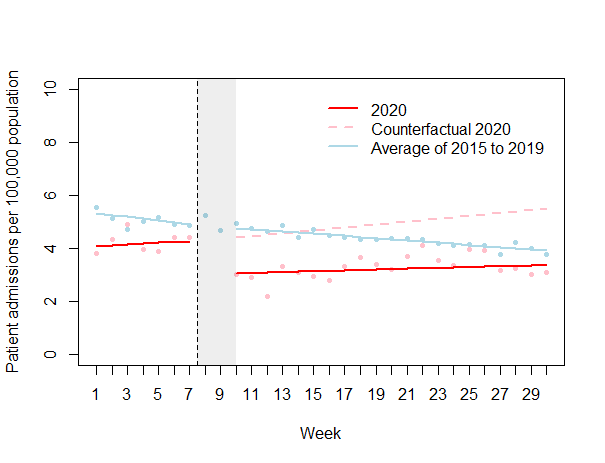 | (b)  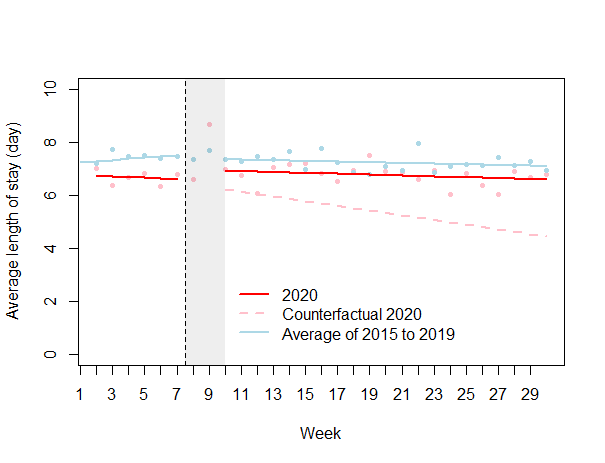 | (c)  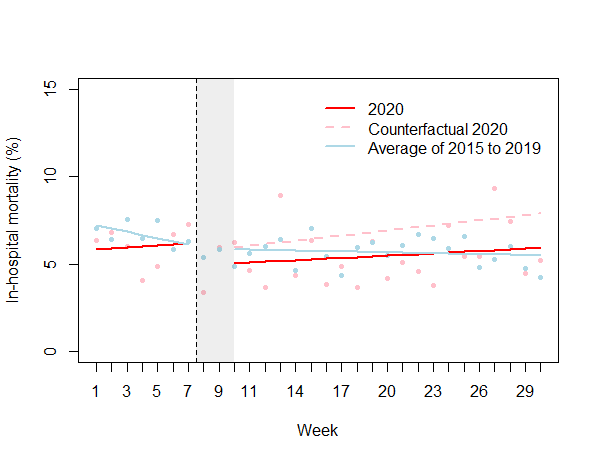 |
| Stroke | | |
| (d)  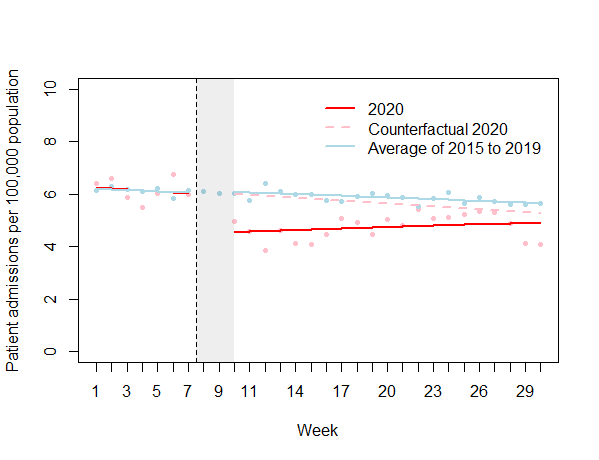 | (e)  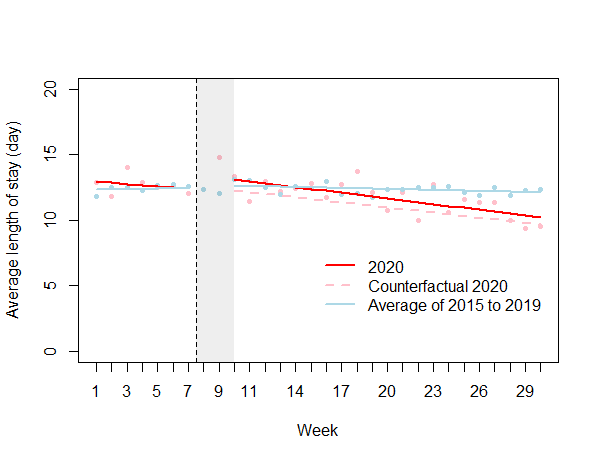 | (f)  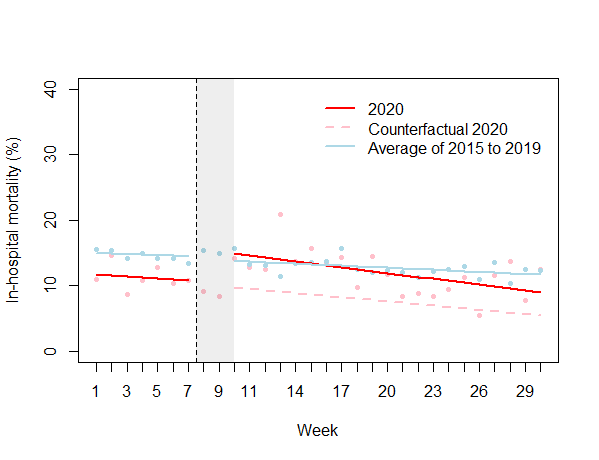 |
| Heart Failure | | |
| (g)  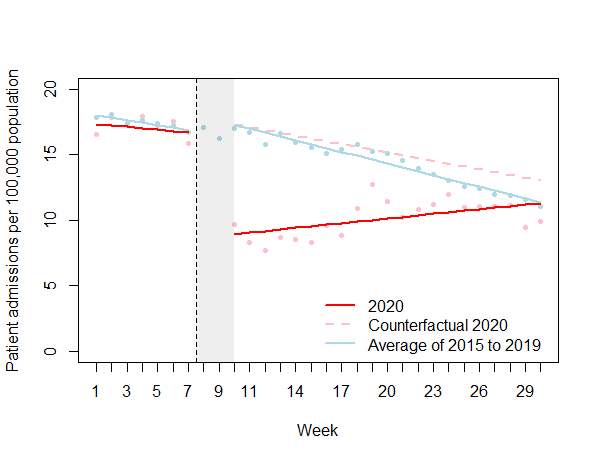 | (h)  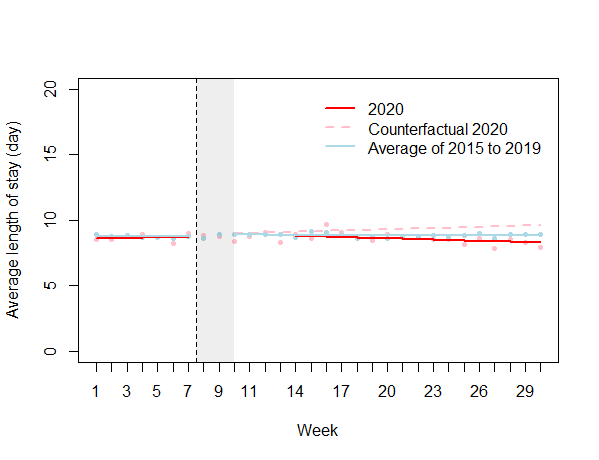 | (i)  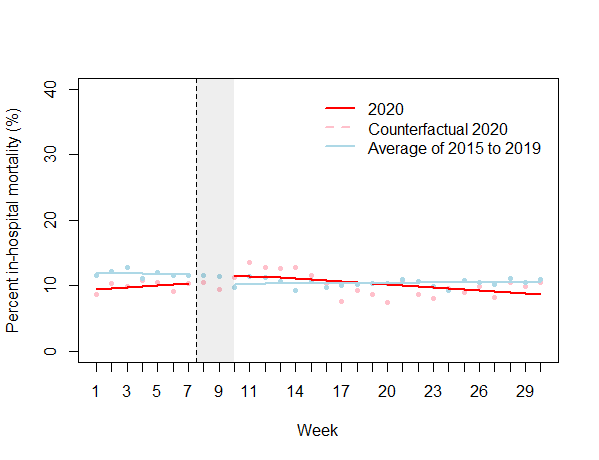 |

**S7 -** Subgroup analyses results of hospitalization rate (/100,000) (a) all inpatient; (b) unplanned; (c) acute myocardial infarction; (d) stroke; (e) heart failure

|  | **Category** | **Level change (95%CI)** |
| --- | --- | --- |
| 1. All Inpatient hospitalization rate (/100,000) | | |
| 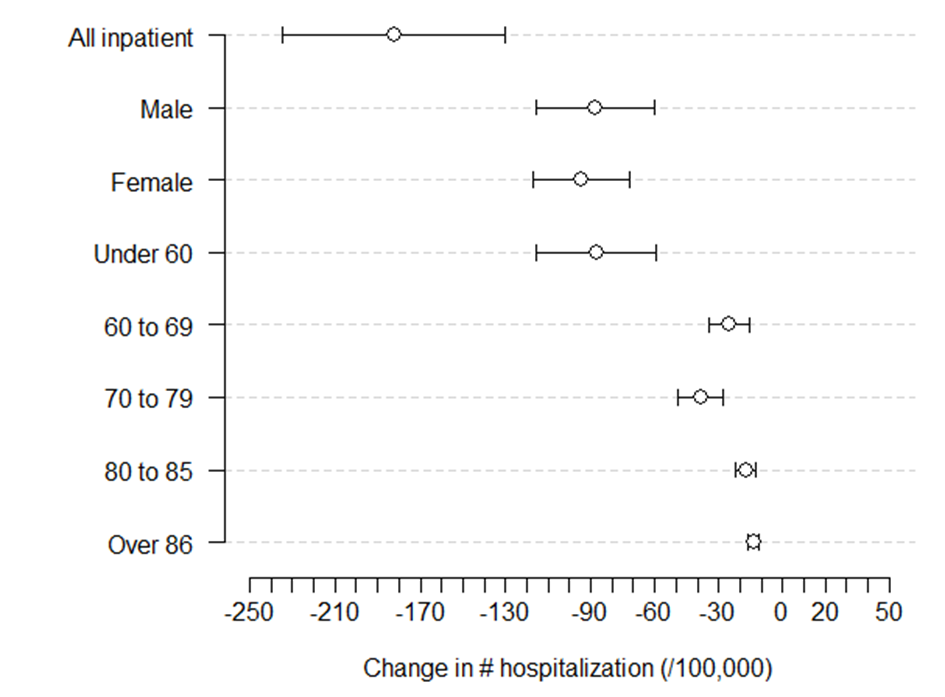 | All | -182 (-235, -130) |
|  | Male | -88 (-116, -60) |
|  | Female | -89 (-117, -61) |
|  | < 60 | -88 (-115, -59) |
|  | 60 – 69 | -25 (-35, -16) |
|  | 70 – 79 | -38 (-49, -28) |
|  | 80 – 85 | -17 (-22, -13) |
|  | >= 86 | -14 (-16, -1) |
|  |  |  |
| 1. Unplanned hospitalization rate (/100,000) |  |  |
| 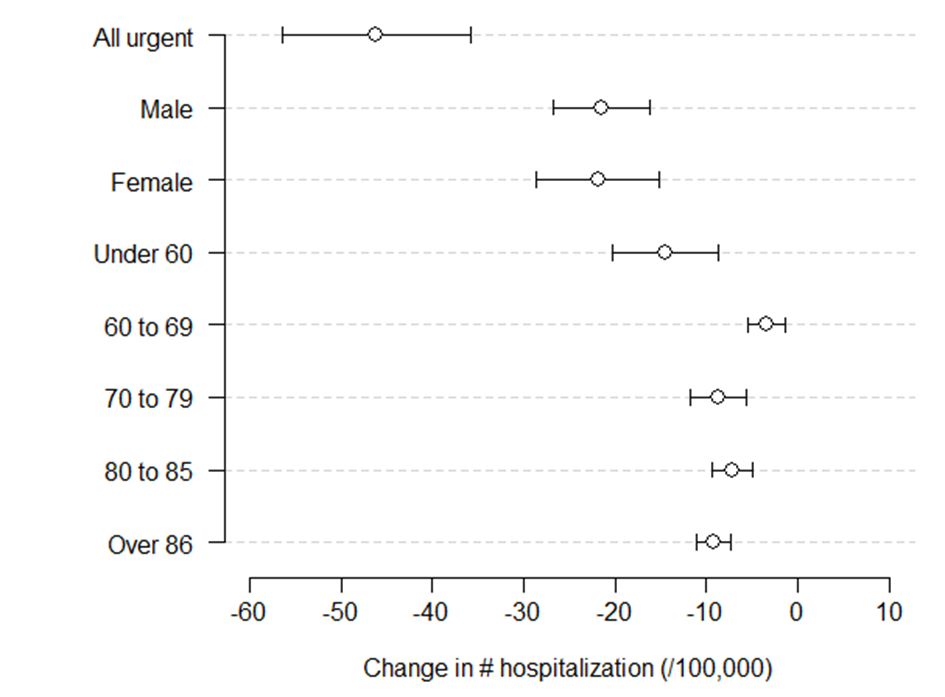 | All | -39 (-51, -26) |
|  | Male | -21 (-27, -14) |
|  | Female | -22 (-29, -15) |
|  | < 60 | -15 (-20, -9) |
|  | 60 – 69 | -3 (-5, -1) |
|  | 70 – 79 | -9 (-12, -6) |
|  | 80 – 85 | -7 (-9, -5) |
|  | >= 86 | -9 (-11, -7) |
|  |  |  |
| 1. AMI hospitalization rate (/100,000) |  |  |
| 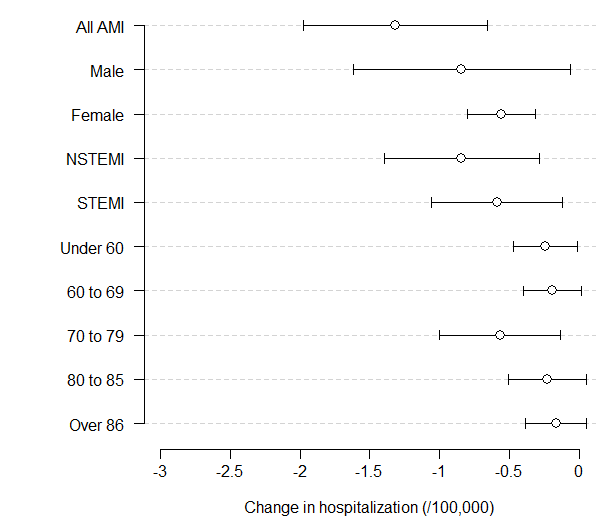 | All | -1.3 (-2.0, -0.7) |
|  | Male | -0.8 (-1.6, -0.06) |
|  | Female | -0.6 (-0.8, -0.3) |
|  | NSTEMI | -0.8 (-1.4, -0.3) |
|  | STEMI | -0.6 (-1.1, -0.1) |
|  | < 60 | -0.2 (-0.5, -0.01) |
|  | 60 – 69 | -0.2 (-0.4, 0.02) |
|  | 70 – 79 | -0.6 (-1.0, -0.1) |
|  | 80 – 85 | -0.2 (-0.5, 0.05) |
|  | >= 86 | -0.2 (-0.4, 0.05) |
|  |  |  |
| 1. Stroke care hospitalization rate (/100,000) |  |  |
| 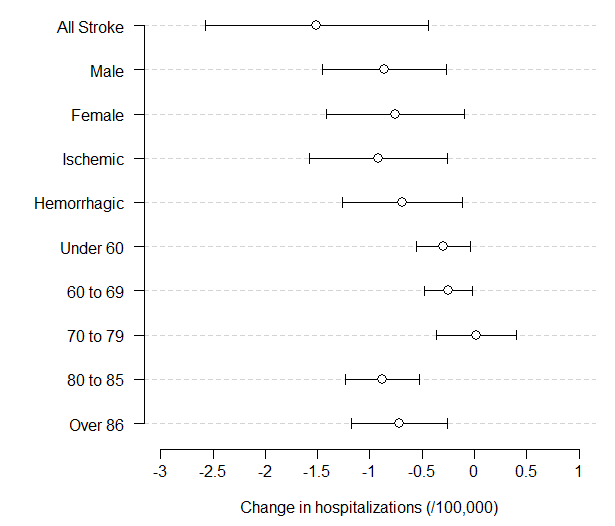 | All | -1.5 (-2.6, -0.44) |
|  | Male | -0.9 (-1.5, -0.3) |
|  | Female | -0.8 (-1.4, -0.09) |
|  | Ischemic | -0.9 (-1.6, -0.3) |
|  | Hemorrhagic | -0.7 (-1.3, -0.11) |
|  | < 60 | -0.3 (-0.6, -0.04) |
|  | 60 – 69 | -0.2 (-0.5, -0.02) |
|  | 70 – 79 | 0.02 (-0.4, 0.4) |
|  | 80 – 85 | -0.9 (-1.2, -0.5) |
|  | >= 86 | -0.7 (-1.2, -0.3) |
|  |  |  |
| 1. Heart failure hospitalization rate (/100,000) |  |  |
| 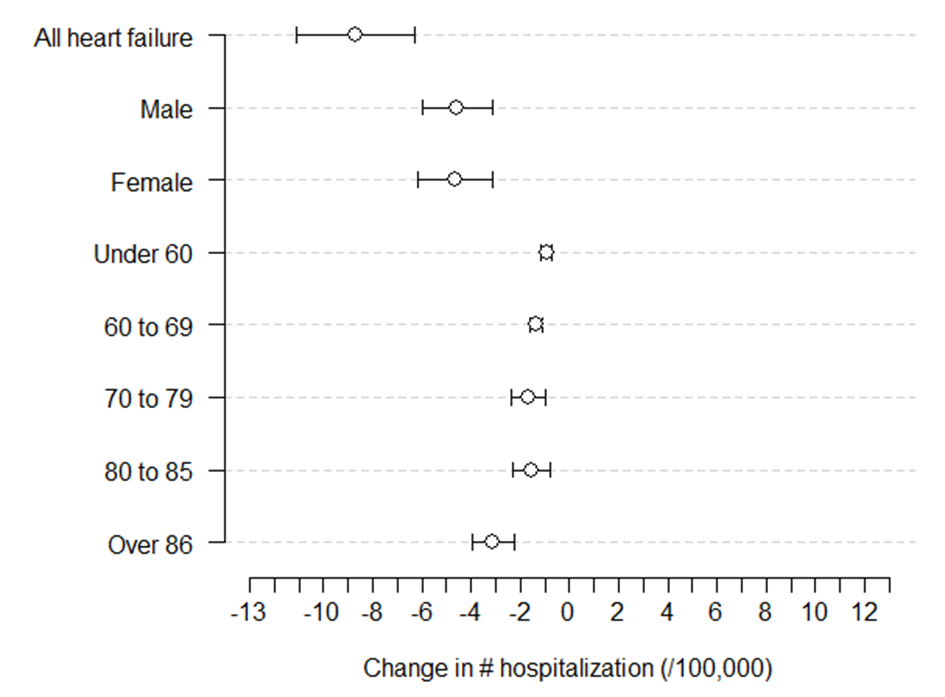 | All | -8.7 (-11.1, -6.3) |
|  | Male | -4.6 (-6.0, -3.1) |
|  | Female | -4.7 (-6.1, -3.2) |
|  | < 60 | -0.9 (-1.1, -0.7) |
|  | 60 – 69 | -1.3 (-1.6, -1.1) |
|  | 70 – 79 | -1.7 (-2.3, -1.0) |
|  | 80 – 85 | -1.6 (-2.3, -0.8) |
|  | >= 86 | -3.1 (-4.0, -2.3) |
